# Correction: Hypoxia Drives Dihydropyrimidine Dehydrogenase Expression in Macrophages and Confers Chemoresistance in Colorectal Cancer

**DOI:** 10.1158/0008-5472.CAN-22-0398

**Published:** 2022-04-04

**Authors:** Marie Malier, Khaldoun Gharzeddine, Marie-Hélène Laverriere, Sabrina Marsili, Fabienne Thomas, Magali Court, Thomas Decaens, Gael Roth, Arnaud Millet

In the original version of this article (1), author Magali Court is mistakenly omitted from the author list. This error has been corrected in the latest online HTML and PDF versions of the article, and the author contributions and disclosures have been updated as necessary. The authors regret this error.
